# Theoretical Insights into Catalytic Mechanism of Protein Arginine Methyltransferase 1

**DOI:** 10.1371/journal.pone.0072424

**Published:** 2013-08-20

**Authors:** Ruihan Zhang, Xin Li, Zhongjie Liang, Kongkai Zhu, Junyan Lu, Xiangqian Kong, Sisheng Ouyang, Lin Li, Yujun George Zheng, Cheng Luo

**Affiliations:** 1 State Key Laboratory of Drug Research, Shanghai Institute of Materia Medica, Chinese Academy of Sciences, Shanghai, China; 2 Division of Nephrology, Shanghai Changzheng Hospital, Shanghai, China; 3 Center for Systems Biology, Soochow University, Jiangsu, China; 4 Department of Chemistry, Program of Molecular Basis of Diseases, Georgia State University, Atlanta, Georgia, United States of America; Universität Stuttgart, Germany

## Abstract

Protein arginine methyltransferase 1 (PRMT1), the major arginine asymmetric dimethylation enzyme in mammals, is emerging as a potential drug target for cancer and cardiovascular disease. Understanding the catalytic mechanism of PRMT1 will facilitate inhibitor design. However, detailed mechanisms of the methyl transfer process and substrate deprotonation of PRMT1 remain unclear. In this study, we present a theoretical study on PRMT1 catalyzed arginine dimethylation by employing molecular dynamics (MD) simulation and quantum mechanics/molecular mechanics (QM/MM) calculation. Ternary complex models, composed of PRMT1, peptide substrate, and S-adenosyl-methionine (AdoMet) as cofactor, were constructed and verified by 30-ns MD simulation. The snapshots selected from the MD trajectory were applied for the QM/MM calculation. The typical S_N_2-favored transition states of the first and second methyl transfers were identified from the potential energy profile. Deprotonation of substrate arginine occurs immediately after methyl transfer, and the carboxylate group of E144 acts as proton acceptor. Furthermore, natural bond orbital analysis and electrostatic potential calculation showed that E144 facilitates the charge redistribution during the reaction and reduces the energy barrier. In this study, we propose the detailed mechanism of PRMT1-catalyzed asymmetric dimethylation, which increases insight on the small-molecule effectors design, and enables further investigations into the physiological function of this family.

## Introduction

Post-transcriptional modifications on basic histone tails, such as methylation, acetylation, and phosphorylation, change the stability of chromatin and affect the binding of transcriptional factors, regulating gene expression without altering the original nucleotide sequence. Histone methylation refers to more than 60 modification enzymes, including modifications introduced by protein lysine methyltransferases (PKMTs) and protein arginine methyltransferases (PRMTs). PRMTs can be classified by their ability to apply asymmetric dimethylation (type I), symmetric dimethylation (type II), or monomethylation (type III), on the N_η_ of arginine guanidino [1]. PRMT1 is the predominant type I arginine methyltransferase in mammals, which transfers two methyl groups from cofactor S-adenosyl-methionine (AdoMet) to the same guanidine nitrogen on substrate arginine. In addition to histone H4R3 [2], the substrates of PRMT1 also include a wide range of non-histone proteins, such as estrogen-receptor (ER) [3], RNA-binding protein TAF15 [4], and PKMT complex component Ash2L [5]. Protein arginine methylation is crucial in gene transcription, mRNA splicing, DNA repair, protein cellular localization, and signaling process. Emerging evidence suggest that the abnormal function of PRMTs is closely associated with the occurrence of cardiovascular diseases and several types of cancer [1]. In detail, global analysis of histone modifications has shown that the dimethylation of histone H4R3 catalyzed by PRMT1 is positively correlated with increasing grades and clinical outcome. Similarly, a recent study has demonstrated that the expression of one of the splice variants of PRMT1 is highly associated with colon cancer and breast cancer. PRMT1 is also essential in mixed lineage leukemia (MLL)-fusion protein-mediated oncogenesis. In addition, PRMT1 may be involved in breast cancer development via the methylation of non-histone substrates, estrogen-receptors (ER). Therefore, the complicated functions of PRMT1 deregulation in diverse cancers provide compelling reasons for understanding the detailed dimethylation mechanism catalyzed by this potential drug target [6]. Small molecular inhibitors targeting PRMTs have been reported, several of which employed structure-based drug design strategy [7,8], reflecting the demand for microscopic understanding of PRMT catalytic mechanism.

Lysine methylation catalyzed by SET-domain containing PKMTs has been studied theoretically. The methyl transfer process is a typical S_N_2 reaction [9], and the methyl accepting nitrogen on lysine must be deprotonated to neutral state by water molecules prior to methyl transfer [10–12]. However, despite the same methyl donor and similar S_N_2 type geometry in the transition state (TS), methylation of arginine seems to be very different from that of lysine. On one hand, because of the stable resonance system in guanidine, arginine is a weaker nucleophile than lysine. The deprotonation of arginine (pKa at approximately 12) is also more difficult than lysine (pKa at approximately 11) in physiological condition, which may result in a different proton transfer mechanism. On the other hand, the AdoMet-binding domain in PRMTs displays higher hydrophobicity compared with the SET domain in PKMTs. In the crystal structure of PRMT1-substrate complex (PDB code: 1 OR 8) [13], no conserved water molecule appears in the active site, indicating that the substrates of PRMT1 are unlikely to be deprotonated by water molecules. However, several polar residues interact with substrate arginine, providing a beneficial reacting condition that varies from PKMTs [13–16]. Experimental studies suggested that arginine methylation catalyzed by PRMTs is due to the proximity effect rather than acid/basic catalysis, and prior deprotonation of guanidino is not essential for methyl transfer [17].

Recently, a theoretical study on the catalytic mechanism of PRMT3 was reported [18], providing a suggestion on the methyl transfer and free energy barrier of reactions by using quantum mechanics/molecular mechanics-molecular dynamics (QM/MM-MD) simulation. However, the sequence of methyl transfer and proton transfer and the charge distribution need further discussion. In addition, as PRMT1 and PRMT3 share a relative low sequence identity, we wonder whether PRMT1, the dominant type PRMT in mammal, adopts the similar catalytic process. In this study, we present a theoretical study by employing molecular dynamics (MD) simulation and quantum mechanics/molecular mechanics (QM/MM) calculation to explore the molecular basis of arginine dimethylation and the proton transfer mechanism. The typical S_N_2-favored transition states of the first and second methyl transfers were identified; the carboxylate group of E144 was determined as proton acceptor. We also analyzed the charge distribution during the reaction, and investigated the order of methyl and proton transfer.

## Materials and Methods

### Simulation System Preparation

The initiating structure of enzyme–substrate-cofactor ternary complex was modeled based on the crystal structure of a rat PRMT1 complex with peptide substrates and S-adenosyl-homocysteine (AdoHcy) (PDB code: 1 OR 8) [13]. The initial conformation of RGG peptide was generated by Discovery Studio v 3.0 (Accelrys Software Inc.) according to Cα position in the crystal structure; the conformation of side-chains were minimized by Amber 10.0 [19] with Cα position restricted. H161 was mutated to tyrosine according to the sequence of human variant (UniProt code: Q99873). Three peptide binding-channels were observed in the complex structure; the one (chain B, RGG) with arginine in the active site was selected as substrate [13]. The PRMT1 N-terminal, which is disordered in 1 OR 8, was constructed based on the crystal structure of CARM1 (PDB code: 3B3F) [20]. We aligned a methyl group from AdoMet complex with DOT1L (PDB code: 3QOW) to AdoHcy in the PRMT1, so that a ternary complex PRMT1-RGG-AdoMet was constructed [21]. Discovery Studio v 3.0 (Accelrys Software Inc.) was applied to add missing hydrogen atoms and minimize the resulting model of PRMT1-RGG-AdoMet complex. PRMT1-meRGG-AdoMet, the reactant for the second methyl transfer, was derived from the product of the first methyl transfer simulation by replacing AdoHcy with AdoMet.

### Molecular Dynamic Simulation

PRMT1 is active under pH 6.0 to 9.25 [17]; thus, we evaluated the protonation state of the residues in the PRMT1-RGG-AdoMet complex at pH 8.0 by H++ program [22]. The amino group on AdoMet (pKa at approximately 9.5) was protonated under specific pH conditions [17]. The covalent and non-bonded parameters of AdoMet were introduced from General Amber force field (GAFF), which is applicable to the simulation of small organic compounds in complexes with biomolecules [23]. Atomic charges of AdoMet and mono-methylated arginine(MRG) were determined using the restrained electrostatic potential (RESP) [24] module in AMBER10.0 [19] at the HF/6-31G* level. The two complexes, PRMT1-RGG-AdoMet and PRMT1-meRGG-AdoMet, were solvated into a cubic box with a 9Å minimum distance between the solute and the edge of the solvent box.

All MD simulations were conducted using AMBER 10.0 [19] with constant temperature and volume periodic boundaries (NVT) after the system was equilibrated at constant temperature and pressure (NPT). Amber99 force field [25,26] for protein and TIP3P model [27] for water were employed. In the MD simulation, the time step used was 2 fs, and the bonds involving hydrogen atoms were constrained by SHAKE [28]. Electrostatic energy was calculated using the Particle Mesh Ewald (PME) method, with a non-bonded cutoff of 8.0 Å. The temperature during the MD simulation was maintained at 300 K by Berendsen control, with a coupling time of 2 ps.

### QM/MM Calculation

**Figure 1:**
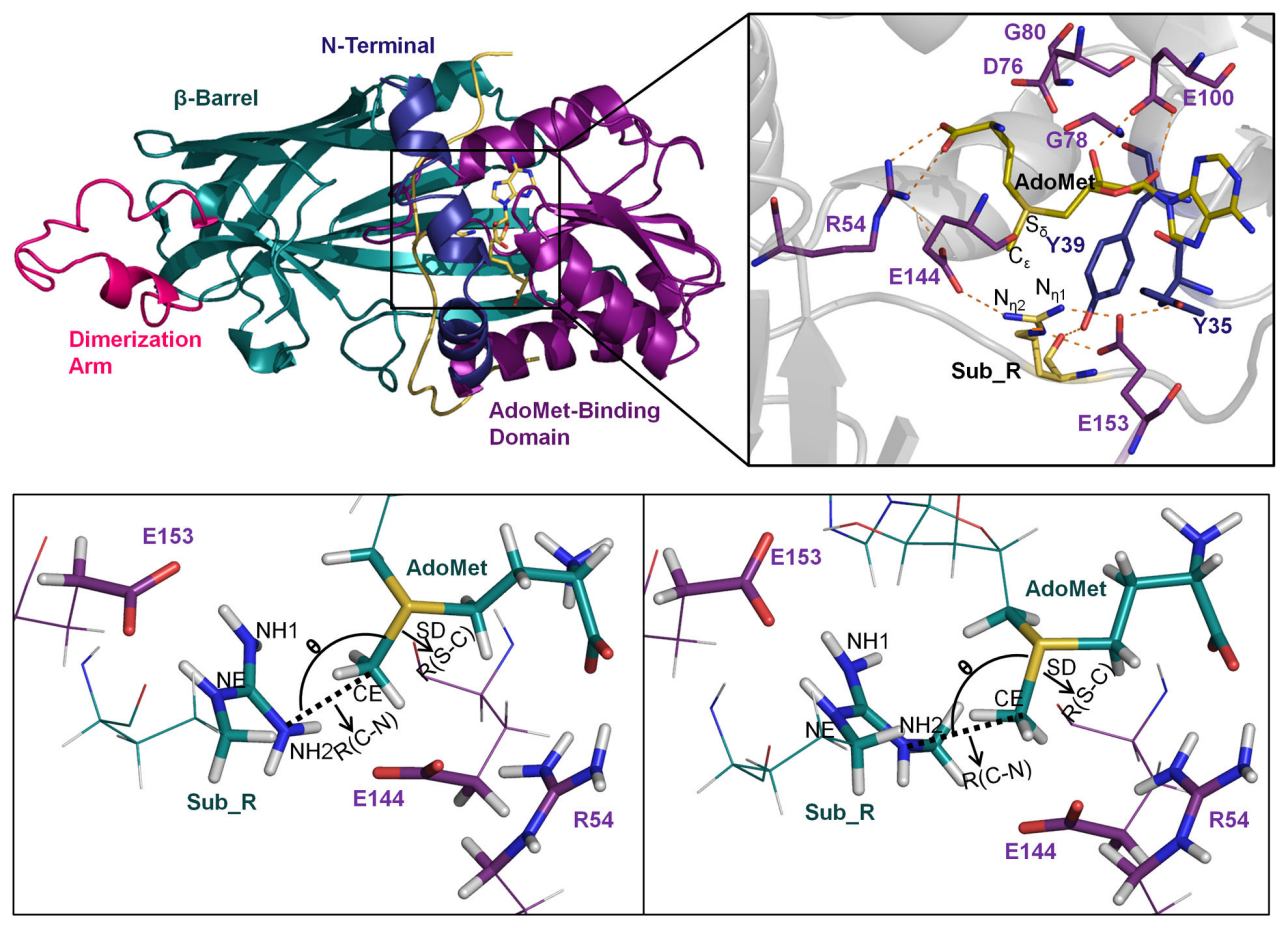
The overall structure of PRMT1-RGG-AdoMet complex and Atoms involved in QM region. Overall structure of (A) the PRMT1-RGG-AdoMet complex and (B) the microenvironment in active site. Atoms involved in the QM region (stick), and the structure parameters of the PRMT1-RGG-AdoMet complex (C) and the PRMT1-meRGG-AdoMet complex (D).

We sampled snapshots from the MD trajectory based on the following criteria: 1) the distance between the C_ε_ of AdoMet and N_η_ of Sub_R is equal to or less than 3.5 Å; 2) the angle between S_δ_, C_ε_, and N_η_ ranges from 150° to 180°; 3) the carboxylate groups of E144 and E153 are within hydrogen bond distance from N_η_ of Sub_R, which is defined as 4.0 Å (Figure 1B). Structures fitting these criteria were extracted from equilibrium MD trajectory, starting at 10 ns, and the interactions in selected snapshots were carefully inspected to ensure its being qualified for subsequent QM/MM calculation.

The sampled snapshots of PRMT1-RGG-AdoMet complex were first minimized with Amber force field encoded in Amber program [19], and then further optimized with QM/MM method implemented in Gaussian 03 package [29]. All QM/MM calculations were performed via the ONIOM method [19,30–33]. The ONIOM method allows a combination of quantum mechanics and molecular mechanics in treatment of a structure, which can be defined as two or three layers with different accuracy, to balance the accuracy and efficiency of computational study. In this study, we defined the catalytic site of the ternary complex as a high layer and the entire system as a low layer, treated by QM and MM, respectively. The QM region, also called the Small Model System (SM), included the methionine part of AdoMet, the guanidino of Sub_R, and most of the polar side-chain of key residues: R54, E144, and E153. SM was treated with density functional (DFT) method [34], with the B3LYP exchange-correlation functional and 6-31G* basis set. The whole system, referred to as the MM region or Real System (R), was described with AmberParm99 force field. Considering that the DFT method usually underestimates the energy barriers of methyl transfer [9,35], we also used the MP2 method with 6-31G* basis set to calculate single-point potential energy of the reactant and the transient. The ONIOM energy was obtained from the following equation [30]:

$${\text{E}}_{\text{ONIOM}}\text{= E}\left(\text{MM,R}\right)\text{+E}\left(\text{QM,SM}\right)\text{-E}\left(\text{MM,SM}\right)$$

Linked hydrogen atoms were employed to saturate heavy atoms of the Real System, which bonded to Model System [36]. Electronic embedding was applied in ONIOM calculation, which incorporates the partial charges of the MM region into quantum mechanical Hamiltonian, so that the electrostatic interaction between QM and MM region as well as the polarization of QM wave function can be better described.

## Results and Discussion

### Structure of the Initial Model

As depicted in the Materials and Methods, the PRMT1-RGG-AdoMet complex model was initially constructed. Structurally, PRMT1 is composed of four parts, namely, N-terminal, AdoMet-binding domain, β barrel, and dimerization arm, as shown in Figure 1A. Although the N-terminal helix αX (residue 1-40) indicates disorder in PRMT1 crystal structures, it is indispensable for cofactor binding and enzymatic activity [13]. Thus, we modeled the structure as mentioned previously. In the final model, the position of motif YFxxY at helix αX was identical to that in other PRMTs [14–16,20], which enables the conserved hydrogen bonds of Y35-E153 and Y39-Sub_R for proper active site organization (Figure 1B). The AdoMet-binding domain was in a Rossmann fold topology, which is a common feature of AdoMet-dependent methyltransferases [37]. The dimerization arm was inserted into the β barrel formed by 10 strands. The reactant for the second methyl transfer, PRMT1-meRGG-AdoMet, was modeled based on the monomethylated product structure optimized by the QM/MM method. Except for the monomethylated arginine, the remaining parts of PRMT1-meRGG-AdoMet retained the identical conformation as the PRMT1-RGG-AdoMet model.

### Stability and Rationality of Complex Models

Although the monomer enzyme was employed in the simulation, the active cavity was presumed steady during computational time scale. Therefore, a 30-ns MD simulation was performed on PRMT1-RGG-AdoMet and PRMT1-meRGG-AdoMet complex to verify the stability of the catalytic center. The root-mean-square deviations (RMSD) of the backbone atoms in the entire system and the distinct domains were calculated based on the initial position. The overall structure of PRMT1-RGG-AdoMet was moderately stable during the simulation, with an RMSD at approximately 4.5 Å, as shown in Figure S2. However, the dimerization arm was very flexible, which largely accounted for the deviation of the entire structure. The deficiency of the first 40 residues in all PRMT1 structures indicates an inherent instability of N-terminal helixes [13,38]. Apart from that, the core region (N-terminal and AdoMet-binding domain) retained almost the same conformation as the inintial frame, and the orientation of key residues was maintained. The PRMT1-meRGG-AdoMet model was as stable as PRMT1-RGG-AdoMet in the 30-ns simulation (Figure S2). Therefore, the structure of these two models was reliable for the following analysis and calculation.

### Microenvironment of Catalysis Center

The hydrogen bond microenvironment is crucial in PRMT enzymatic activity. The orientations of the key residues in the active site were further analyzed in detail. The hydrogen bonds among AdoMet, Sub_R, and PRMT1 remained stable during the MD simulation progress (Table S1, Figure S1). The hydrogen bond between E144 carboxyl oxygen and Sub_R N_η2_ orients the guanidino group in a direction that faces the AdoMet methyl group, as shown in Figure 1B. In the consideration of E153, in PRMT1 crystal structure (PDB code: 1 OR 8) solved under a low pH condition (approximately 4.7), the protonated E153 is less sufficient in the electrostatic interaction with positive-charged arginine in the substrate, comparing with deprotonated E153 [13]. In our PRMT1-RGG-AdoMet complex model, whose protonation state was evaluated in pH 8.0, E153 was deprotonated and adopted the same direction as its counterpart in PRMT3 [15] and CARM1 [20], thereby forming a hydrogen bond with N_η1_ or N_δ_ of Sub_R. Both glutamates, E144 and E153, are conserved among the PRMT family, and their indispensable roles for PRMT1 catalysis have been verified by mutation experiments [17]. R54, another conserved residue among PRMTs, formed hydrogen bonds with AdoMet amino group and E144 carboxylate to maintain the organization of the catalytic center. The water occupancy in active site calculated by VMD 1.9.1 [39] suggested during our MD simulation, unlike in GLP (PDB code: 3HNA) [40], there are no water molecules in the active site forming steady hydrogen bond with substrate guanidino, which indicated that water molecules are not directly involved in reaction (Figure S3). In conclusion, the hydrogen-bonding net among AdoMet, Sub_R, E144, E153, and R54 are important to guarantee the geometry of the active site, and their stability during the MD simulation ensured the reliability of the modeling complex for subsequent calculations.

### Structure Parameters of Snapshots from MD trajectory

The atoms involved in the QM region of PRMT1-RGG-AdoMet and PRMT1-meRGG-AdoMet are shown in Figure 1C and 1D, respectively. We defined three structural parameters, including the distance between CE (AdoMet) and NH2 (Sub_R) as R(C–N), the distance between SD (AdoMet) and CE (AdoMet) as R(S–C), and the angle of NH2-CE-SD as θ (Figure 1C and 1D). Among which, R(C–N) and θ are the definitive factors of an S_N_2-favored structure. We investigated the distribution of these structural parameters during the MD simulation of PRMT1-RGG-AdoMet and PRMT1-meRGG-AdoMet by sampling every 10 ps to further validate the reliability of our sampling strategy and explore the influence of the additional substrate methyl group on the active site (Figure S4). For the PRMT1-RGG-AdoMet model, the snapshots adopted conformation with 4.3 Å for average R(C–N) and 150.3° for average θ. PRMT1-meRGG-AdoMet model had a broad distribution of θ, with an average value of 135.5°, whereas R(C–N) remained at approximately 4.5 Å. On one hand, these data indicated the reliability of the sampling processes, in which the active site maintained an S_N_2-allowed alignment during the 30-ns simulation. On the other hand, the difference of the θ distributions between these two models reflected the influence of the additional methyl group on the geometry of the active site. For the same reason, the chance to sample an eligible PRMT1-meRGG-AdoMet structure for QM/MM calculation decreased during the MD simulation. It implicated that the second methyl group might transfer right after the first one, as suggested in previous kinetic experiment that PRMT1 catalyzes substrate dimethylation in a partially processive manner [41]. In PRMT1-meRGG-AdoMet, the methyl group on Sub_R-NH2 adopted a “downward” conformation to avoid hindrance with other parts of guanidino, blocking the space between guanidino and E144. This conformation of Sub_R increased the difficulty in forming hydrogen bonds between OE2(E144) and NH2(Sub_R), and resulted in the position flexibility of NH2, demonstrated as a broad distribution of angle θ. This study indicates that E144 contributes to correcting the direction of methyl accepting nitrogen to guarantee S_N_2-favored in-line geometry.

### Mechanism of PRMT1 Catalyzed Arginine Methylation

#### Methyl Transfer Process

**Figure 2:**
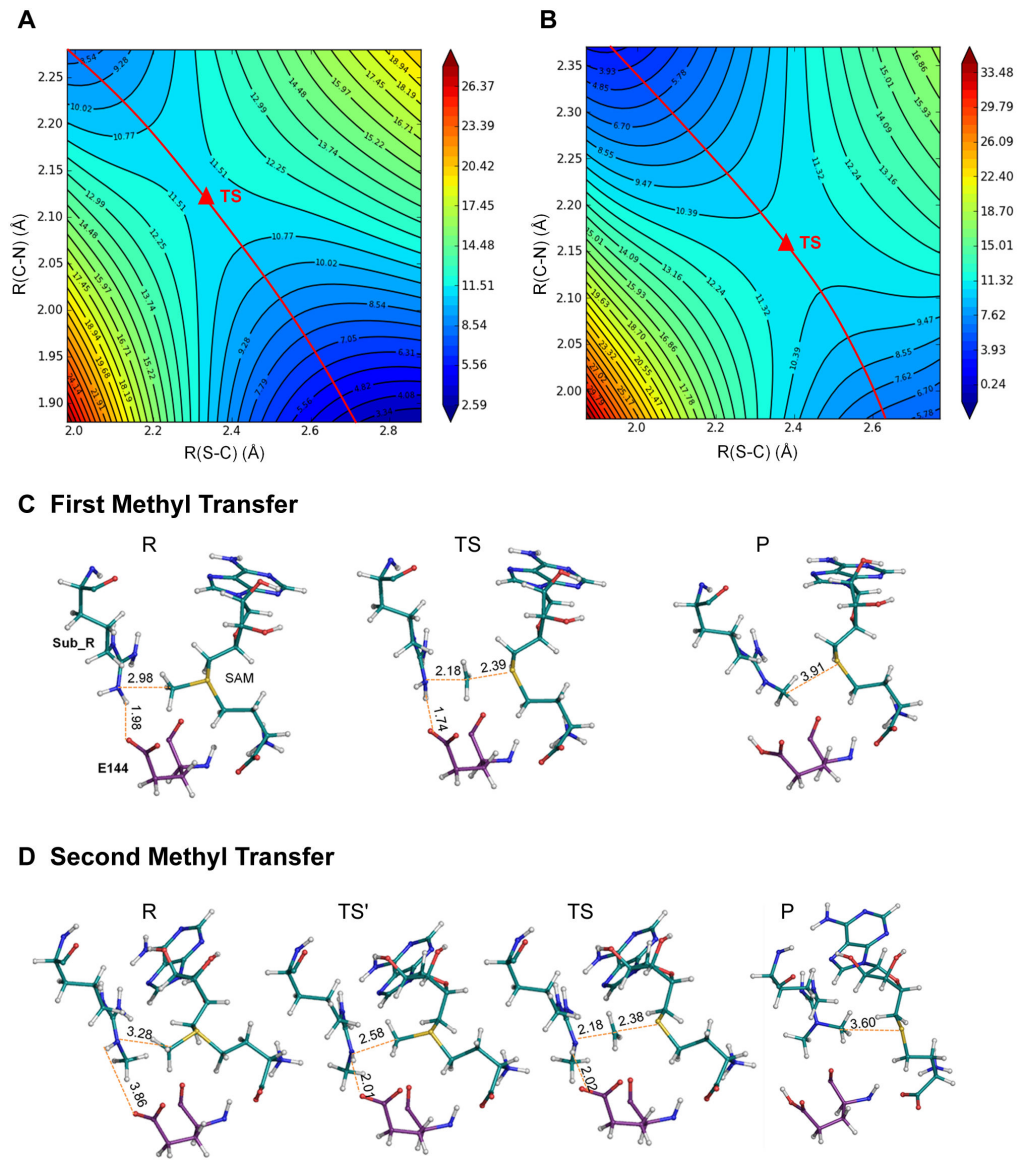
Potential Energy Surface of the first (A) and second (B) methyl transfer. Only the states adjacent to TS were included in the contour plot. Structure of the reactant (R), S_N_2 transition state (TS), and product (P) in the first (C) and second (D) methyl transfers.

The QM region shown in Figure 1 was composed of the atoms involved in the methyl transfer and adjacent polar interactions that facilitate the reaction (parts of AdoMet, Sub_R, R54, E144, and E153). Potential energy profile during the methyl transfer process was calculated using the DFT method. The conformation of the reactant and product was identified from the potential energy curve plotted as a function of R(C–N) (Figure S5). The TS structure and energy were determined from two-dimensional potential energy surface by defining R(C–N) and R(S–C) as the reaction coordinates (Figure 2). The structures of the reactant, TS, and product in two reaction steps are extracted and shown in Figure 2. For the reactant state, the distance parameters reflected a more compact active site with apo substrate than with monomethylated substrate. This condition may be related to the increased steric hindrance induced by the methyl group. Despite their different reactant structures, the TS parameters of the first and second methyl transfer were almost the same: R(C–N) = 2.18 Å and R(S–C) = 2.39 Å and 2.38 Å, respectively. The structure parameters of distance R(C–N) and angle θ in reactant and TS for both models corresponded to that of PKMTs [10,42] (Table 1). In the earlier reported work on PRMT3, R(C–N) and R(S–C) for the first methyl transfer are 2.2 Å and 2.0 Å, respectively; for the second methyl transfer are 2.3 Å and 2.1 Å [18]. It indicated a similar S_N_2-favored geometry in transient states of PRMTs. The value of angle θ in TS indicated an acceptable alignment of reactive atoms which satisfied the requirement of S_N_2 attack. For the first methyl transfer, OE2(E144) remained near NH2(Sub_R) and maintained a stable hydrogen bond from the initial frame to the end frame. However, in the reactant structure for the second methyl transfer, OE2 and NH2 were relatively distant from each other, and the additional methyl group on NH2 blocked the hydrogen bond interaction between OE2 and NH2. Therefore, a small energy barrier was observed before TS, as demonstrated in the potential energy curve of the second methyl transfer (Figure S5). This belonged to the E144 side-chain flipping, which enabled the formation of hydrogen bonds between OE2 and NH2.

**Table 1:** Potential Energy Barrier and Geometric Parameters in S_N_2 Transition State^*^.

	**Reactant**		**Transient**		**ΔE**	
	R(C–N)	θ	R(C–N)	θ	B3LYP	MP2
	Å	°	Å	°	Kcal/mol	Kcal/mol
1st Methyl Transfer	2.98	157.8	2.18	172.1	11.76	19.08
2nd Methyl Transfer	3.28	118.8	2.18	177.8	11.63	14.94

Although DFT method enables us to obtain reliable optimized structures, it may underestimate the energy barrier of S_N_2 methyl transfer reaction [9,35]. Thus, we also employed the MP2 method to obtain the potential energy barrier by calculating the single point energy of the reactant and TS. The results in Table 1 indicated that the second methyl transfer might be faster than the first, which follows the result of enzyme kinetic test: the experimental rate constant K_cat_ for apo and monomethylated substrate are 0.39 min^-1^ and 0.79 min^-1^, respectively [43]. The same conclusion was obtained by QM/MM-MD study of PRMT3 [18]. For Rubisco LSMT, an enzyme with lysine dimethylation activity, the K_cat_ for apo and monomethylated substrate are 0.0033 min^-1^ and 0.015min^-1^, respectively, which also reflects a slightly faster reaction rate for the second step methylation [44]. In the theoretical study of Rubisco LSMT, the potential energy barriers for the first and second methyl transfers calculated by MP2/6-31+G(d, p)//MM were 21.4 kcal/mol and 19.6 kcal/mol [45]. Therefore, both the computational and experimental results indicated a more efficient reaction catalyzed by PRMT1 than Rubisco LSMT. Although arginine is a weaker nucleophile than lysine, the methylation rate of the former was probably faster than the latter. This conclusion suggested that certain facilitating factors must be involved in PRMT1 active site to accelerate the reaction. In addition, although the potential energy barrier of the second methyl transfer was lower than the first, the value of experimental Michaelis constant K_m_ reflects a relatively lower binding affinity of methylated substrates in the catalytic center than that of the apo substrate [43,46].

**Figure 3:**
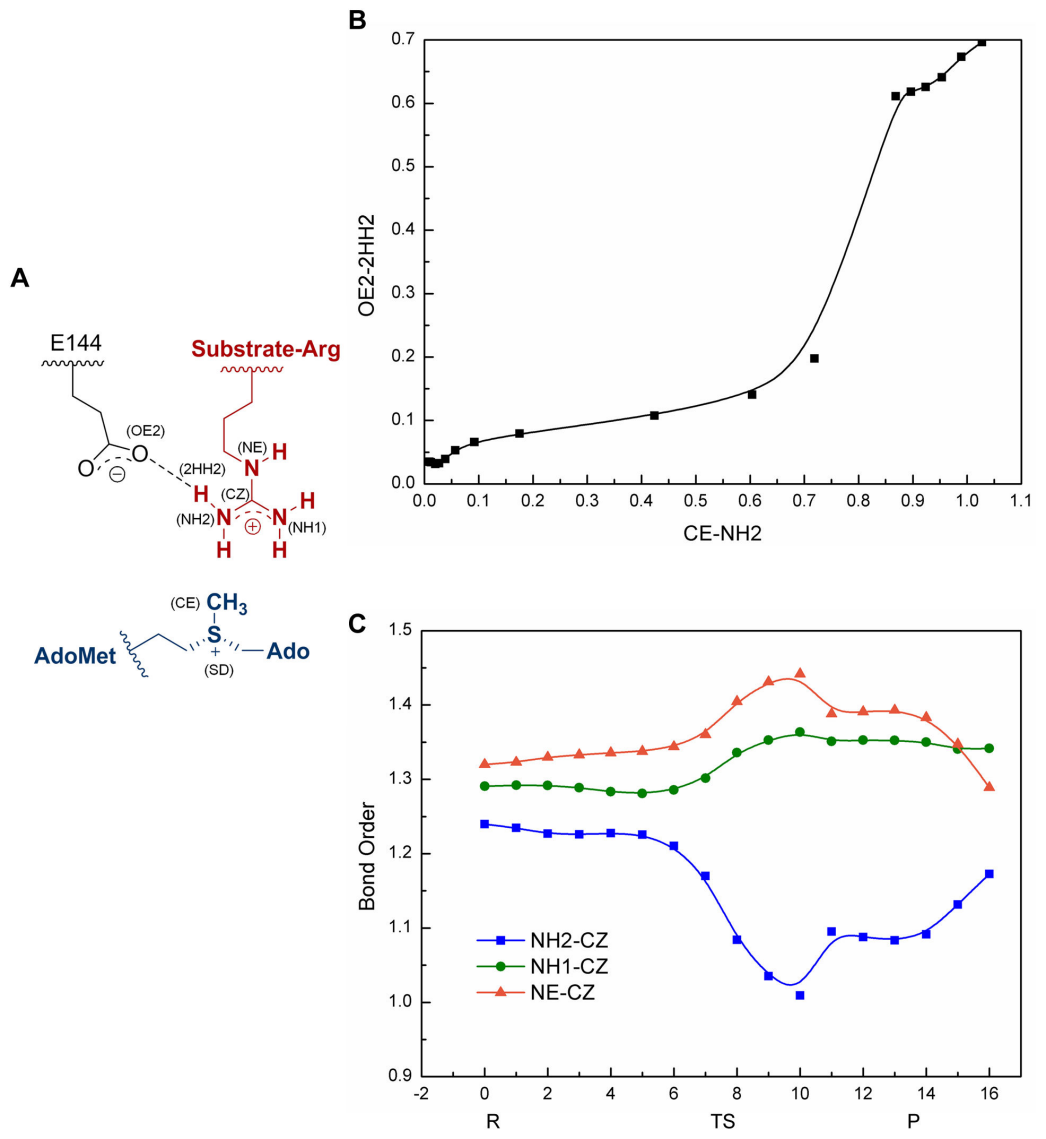
Evolution of the Wiberg bond order during the first methyl transfer. (A) Illustration of the bond and atom name. (B) The relationship between the formation of OE2-2HH2 and CE-NH2 suggests that deprotonation occurs after methyl transfer. (C) The bond order evolution involved in the guanidino group indicates the charge redistribution during reaction (R: Reactant, TS: S_N_2 transition state, P: product)..

Natural Bond Order (NBO) analysis encoded in Gaussian 03 [29] was performed to obtain Wiberg bond order [9,47] diagram for further understanding the methyl transfer mechanism and explore why the second methyl transfer could be faster than the first one. In substrate arginine, guanidino cation is stabilized via efficient resonance. Thus, the N_η_ atoms can be considered as between the sp2 and sp3 hybridization state. During reaction process, the bond order of NH2-CZ gradually decreased to 1 in TS, as shown in Figure 3C. This result is in accordance with the fact that the lone pair on sp3 nitrogen has improved nucleophilicity over π electrons on sp2 nitrogen. The bond order of NH2-CZ in the product was 0.1 smaller than that in the reactant, indicating a more sp3-like NH2 for the next step reaction. We hypothesized that the resonance system of monomethylated guanidino became less efficient after the first methyl transfer. Therefore, nitrogen NH2 was closer to sp3 state in the second reactant, resulting in the second methyl transfer being faster than the first.

### Proton Transfer Mechanism

The experiment of solvent isotope effects (SIE) suggested that no prior substrate deprotonation is required for PRMT1 catalysis [17]. The following theoretical analysis based on QM calculations was performed to investigate the proton transfer process involved in PRMT1 catalyzed arginine methylation. Confirmations extracted from the potential energy profile showed that the guanidino group was deprotonated immediately after methyl transfer, and the proton may transfer to the acid oxygen on E144 (Figure 2C). This result is in accordance with the study on PRMT3, which proposed the proton transfers to E326, the counterpart of E144 in PRMT3 [18]. Thus, the important role of conserved glutamine in PRMT catalysis is revealed by these two theoretical investigations. NBO analysis was performed to explore the precedence relationship between methyl transfer and deprotonation. In the Wiberg bond order diagram (Figure 3B), the concave shape of the line demonstrated that the formation of bond between OE2 (E144) and 2HH2 (Sub_R) occurred after the formation of the bond between CE (AdoMet) and NH2 (Sub_R), or the proton transfer next to methyl transfer [9].

**Figure 4:**
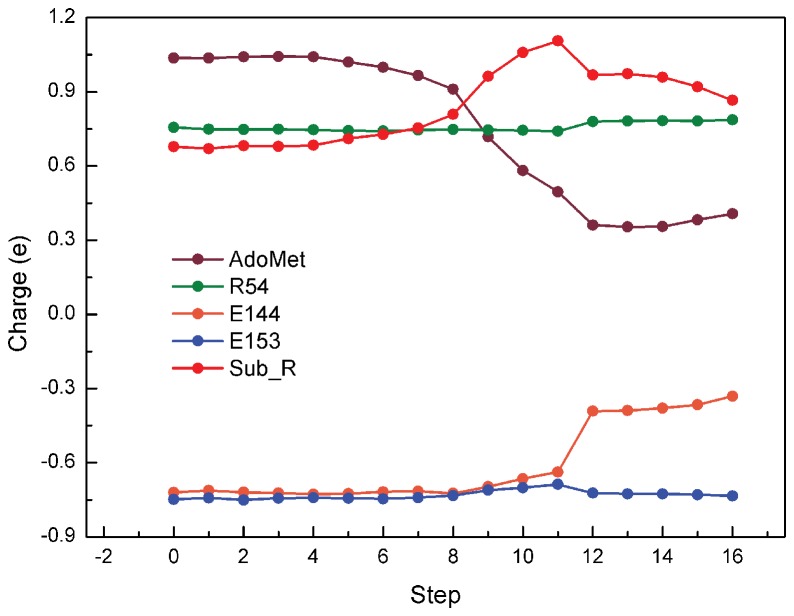
Evolution of electrostatic potential (ESP) charge distribution during the first methyl transfer. (R: Reactant, TS: S _N_2 transition state, P: product).

We analyzed the evolution of electrostatic potential (ESP) in the QM region for the first methyl transfer process to further understand the deprotonation of NH2. Charges on R54 and E153 remained constant during the reaction, whereas charges on Sub_R, AdoMet, and E144 showed apparent variations, suggesting these three residues were involved in reaction process, as demonstrated in Figure 4. As expected, positive charges on AdoMet obviously decreased during the reaction, which indicated the methyl group leaving off the sulfur atom, whereas the positive charge on Sub_R only increased slightly in the product state. This result is likely due to the interference of the additional methyl group on guanidino resonance system, which abates the delocalization of the charges. However, a sudden downward fluctuation was observed on the Sub_R curve, corresponding to the upward fluctuation of the E144 curve. This result suggested that the unstable aggregating positive charge on Sub_R in TS was largely relieved by proton transferring to E144.

## Conclusion

**Figure 5:**
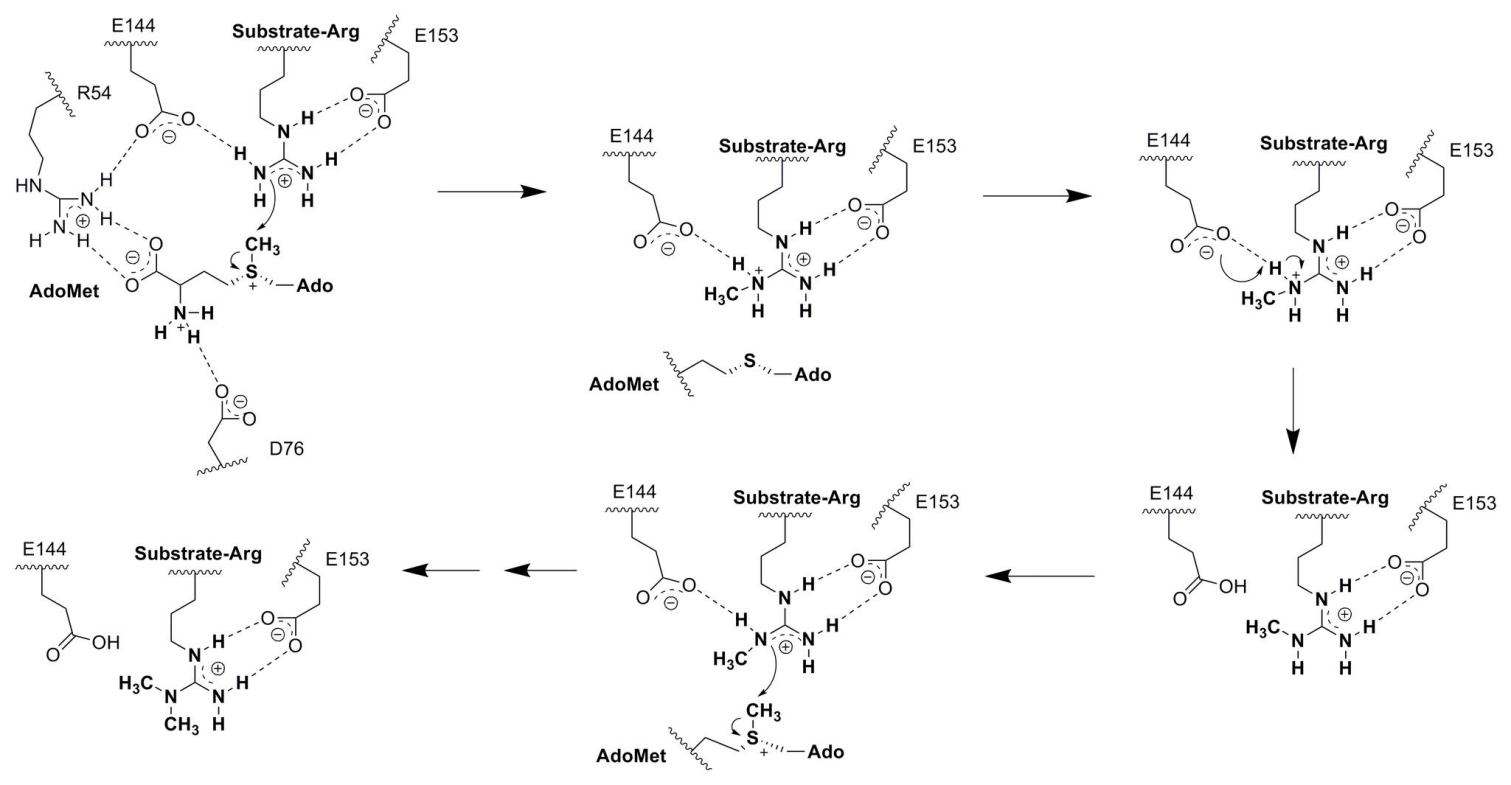
Proposed PRMT1 Catalytic Mechanism.

The present study revealed the mechanism of methyl transfer reaction catalyzed by arginine methyltransferase PRMT1 via theoretical computation (Figure 5). A model of PRMT1-substrate-cofactor complex was constructed, and a 30-ns MD simulation was performed to ensure the stability and rationality of the subsequent calculations. Optimized conformations extracted from the MD trajectory were applied in the QM/MM study. The potential energy profile was plotted, revealing the transition of the structure and energy during reaction. The TS conformation of both reaction steps extracted from the two-dimension potential energy surface displayed the typical geometry required by the S_N_2 reaction. The potential energy barriers of the two-step reactions calculated by MP2/6-31G* revealed that the second methyl transfer might be faster than the first. Through NBO and ESP analysis, we discovered the importance of E144: orienting methyl accepting nitrogen, facilitating nucleophilic attack, reducing TS potential energy, and accepting substrate proton. E144 forms a hydrogen bond with the reactive nitrogen on guanidino, helping to redistribute the aggregated positive charge during methyl transfer.

Arginine is weaker than lysine in nucleophilic attacking because the electrons on guanidino are partially delocalized rather than purely lone pair. Therefore, methylation of arginine requires more assistance to enhance the nucleophilicity of guanidino. R54, E144, and E153 are highly conserved residues in PRMTs [14,15,20,48,49], and their fixed positions and interacting patterns in the active site indicated the indispensability of these residues for protein arginine methylation. In this computational study, we discussed the importance of E144 in PRMT1 catalysis. In summary, we provide a detailed hypothesis of arginine asymmetric dimethylation catalyzed by PRMT1 and discuss the charge distribution and proton transfer process in detail. However, the catalytic mechanism of PRMTs requires further exploration to answer certain questions, such as those on product specificity [50]. Further understanding the PRMT1 catalytic mechanism will be beneficial for the rational design of inhibitors with both efficiency and specificity.

## Supporting Information

Figure S1 **Conserved hydrogen bonds during MD simulation.**
(TIF)

Figure S2 **Root-mean-square deviations (RMSD) of PRMT1-RGG-AdoMet (A) and PRMT1-meRGG-AdoMet (B) during 30-ns MD simulation.** Core: Core Region (Active Site); Arm: Dimerization Arm; All: Entire Structure.
(TIF)

Figure S3 **Water occupancy in active site during MD simulation (A).** Yellow meshes represent the position of water occupancy higher than 50%.
(TIF)

Figure S4 **Distribution of reaction parameters during MD simulation.** A: PRMT1-RGG-AdoMet complex. B: PRMT1-meRGG-AdoMet complex.
(TIF)

Figure S5 **Potential energy curve of the first and second methyl transfer obtained by defining the distance of R(C–N) as the reaction coordinate.**
(TIF)

Table S1 **The occupancy of key hydrogen bonds during 30-ns MD simulation performed on PRMT1-RGG-AdoMet model.**
(DOC)
