# Supplementary figures and images for: Theoretical Insights into Catalytic Mechanism of Protein Arginine Methyltransferase 1
Source: PLoS One. 2013 Aug 20;8(8):e72424. doi: 10.1371/journal.pone.0072424 (PMC3748068; doi:10.1371/journal.pone.0072424)

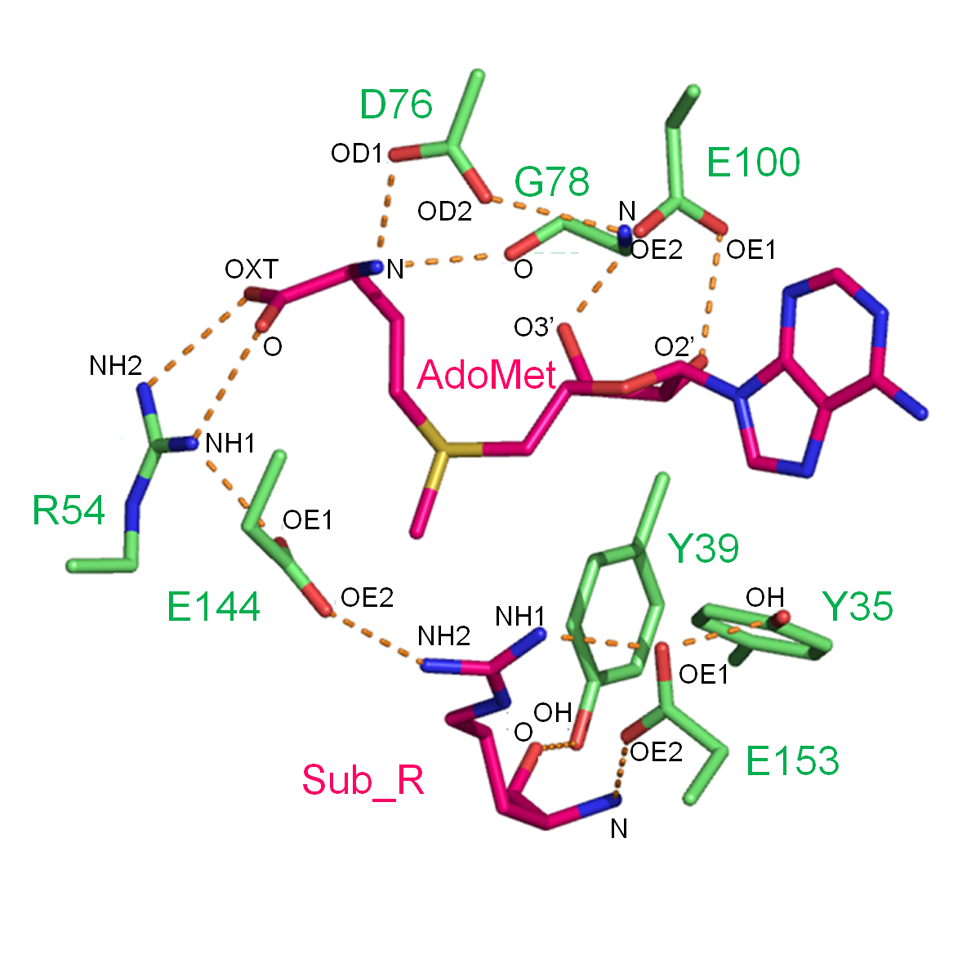

Supplement: Figure S1 — Conserved hydrogen bonds during MD simulation. (TIF) [file pone.0072424.s001.tif]

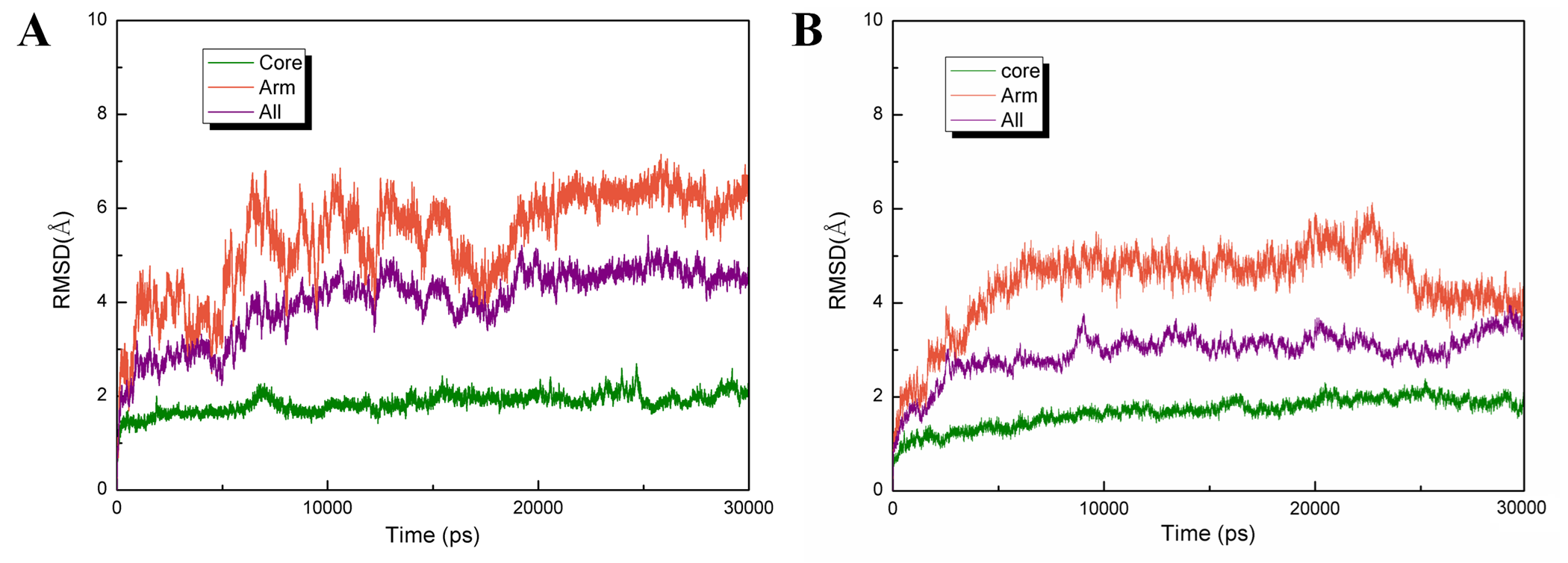

Supplement: Figure S2 — Root-mean-square deviations (RMSD) of PRMT1-RGG-AdoMet (A) and PRMT1-meRGG-AdoMet (B) during 30-ns MD simulation. Core: Core Region (Active Site); Arm: Dimerization Arm; All: Entire Structure. (TIF) [file pone.0072424.s002.tif]

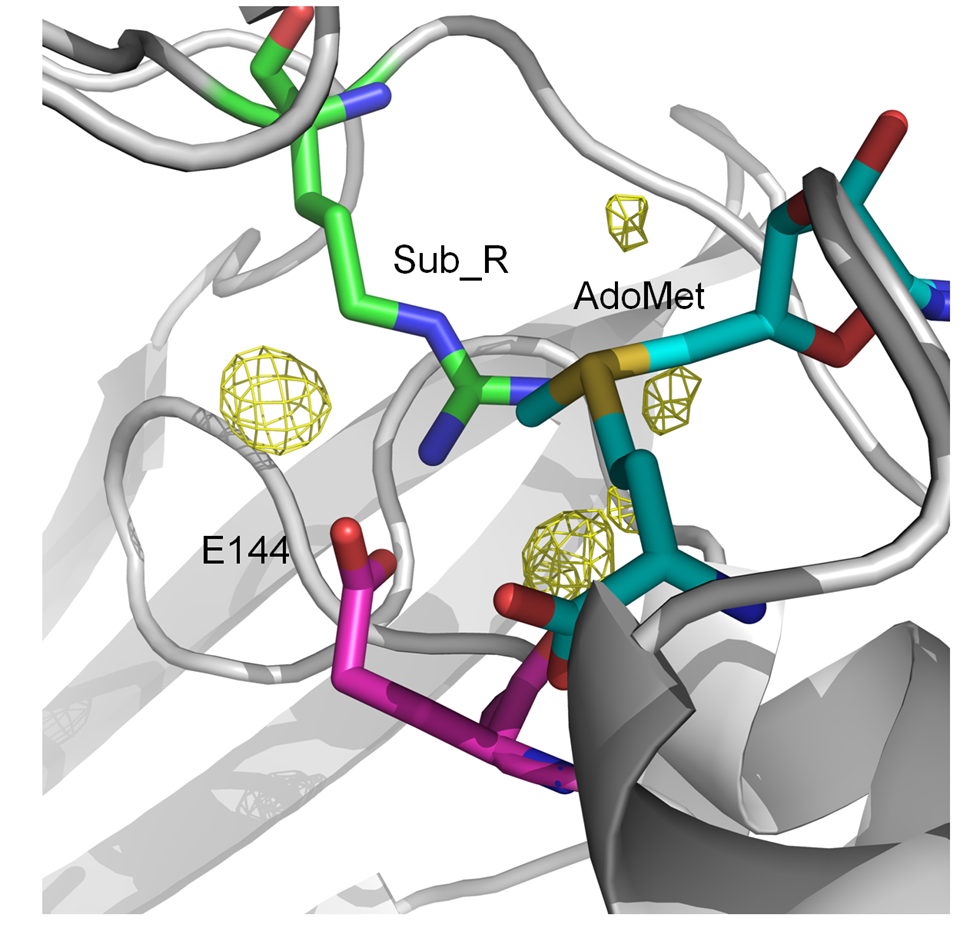

Supplement: Figure S3 — Water occupancy in active site during MD simulation (A). Yellow meshes represent the position of water occupancy higher than 50%. (TIF) [file pone.0072424.s003.tif]
