# Supplementary material for: Theoretical Insights into Catalytic Mechanism of Protein Arginine Methyltransferase 1
Source: PLoS One. 2013 Aug 20;8(8):e72424. doi: 10.1371/journal.pone.0072424 (PMC3748068; doi:10.1371/journal.pone.0072424)
Supplement: Table S1 — The occupancy of key hydrogen bonds during 30-ns MD simulation performed on PRMT1-RGG-AdoMet model. (DOC) [file pone.0072424.s006.doc]

**Supporting Information Table S1.**

The occupancy of key hydrogen bonds during 30-ns MD simulation performed on PRMT1-RGG-AdoMet model^*^

| donor | acceptor | occupancy% | function^**^ | | | |
| --- | --- | --- | --- | --- | --- | --- |
|  |  |  | S | R | C | O |
| E100@OE1 | AdoMet@O3' | 97.46 |  |  |  |  |
| E100@OE1 | AdoMet@O2' | 95.15 |  |  |  |  |
| E100@OE2 | AdoMet@O3' | 71.48 |  |  |  |  |
| E100@OE2 | AdoMet@O2' | 99.96 |  |  |  |  |
| G78@O | AdoMet@N | 39.51 |  |  |  |  |
| D76@OD1 | AdoMet@N | 31.62 |  |  |  |  |
| D76@OD2 | AdoMet@N | 23.96 |  |  |  |  |
| AdoMet@OXT | R54@NH2 | 74.01 |  |  |  |  |
| AdoMet@O | R54@NH1 | 61.33 |  |  |  |  |
| AdoMet@O | R54@NH2 | 50.99 |  |  |  |  |
| E153@OE1 | Sub_R@NE | 39.17 |  |  |  |  |
| E144@OE1 | Sub_R@NH1 | 29.49 |  |  |  |  |
| E144@OE2 | Sub_R@NH1 | 18.76 |  |  |  |  |
| Sub_R@O | Y39@OH | 16.42 |  |  |  |  |
| E144@OE1 | Sub_R@NH2 | 38.91 |  |  |  |  |
| E144@OE2 | Sub_R@NH2 | 33.10 |  |  |  |  |
| E153@OE2 | Sub_R@NH1 | 79.34 |  |  |  |  |
| E153@OE1 | Sub_R@NH1 | 64.84 |  |  |  |  |
| E153@OE2 | Sub_R@NE | 46.75 |  |  |  |  |
| E144@OE2 | AdoMet@N | 39.87 |  |  |  |  |
| E144@OE1 | R54@NH1 | 57.98 |  |  |  |  |
| E144@OE2 | R54@NH1 | 50.41 |  |  |  |  |
| E153@OE1 | Y35@OH | 39.18 |  |  |  |  |
| E153@OE2 | Y35@OH | 35.93 |  |  |  |  |

^*^Distance and angle cutoff of hydrogen bond was defined as 4.0Å and 0°.

^**^S=AdoMet Binding R=Sub_R Binding C=Catalysis O=Residues Orientation
